# Lipid measures for prediction of incident cardiovascular disease in diabetic and non-diabetic adults: results of the 8.6 years follow-up of a population based cohort study

**DOI:** 10.1186/1476-511X-9-6

**Published:** 2010-01-23

**Authors:** Maryam Tohidi, Masumeh Hatami, Farzad Hadaegh, Maryam Safarkhani, Hadi Harati, Fereidoun Azizi

**Affiliations:** 1 Prevention of Metabolic Disorders Research Center, Research Institute for Endocrine Sciences, Shahid Beheshti University of Medical Sciences, Yaman street, Velenjak, Tehran, Iran; 2 Endocrine Research Center, Research Institute for Endocrine Sciences, Shahid Beheshti University of Medical Sciences, Yaman street, Velenjak, Tehran, Iran

## Abstract

**Background:**

Diabetes is a strong risk factor for cardiovascular disease (CVD).The relative role of various lipid measures in determining CVD risk in diabetic patients is still a subject of debate. We aimed to compare performance of different lipid measures as predictors of CVD using discrimination and fitting characteristics in individuals with and without diabetes mellitus from a Middle East Caucasian population.

**Methods:**

The study population consisted of 1021 diabetic (men = 413, women = 608) and 5310 non-diabetic (men = 2317, women = 2993) subjects, aged ≥ 30 years, free of CVD at baseline. The adjusted hazard ratios (HRs) for CVD were calculated for a 1 standard deviation (SD) change in total cholesterol (TC), log-transformed triglyceride (TG), high density lipoprotein cholesterol (HDL-C), low density lipoprotein cholesterol (LDL-C), non-HDL-C, TC/HDL-C and log-transformed TG/HDL-C using Cox proportional regression analysis. Incident CVD was ascertained over a median of 8.6 years of follow-up.

**Results:**

A total of 189 (men = 91, women = 98) and 263(men = 169, women = 94) CVD events occurred, in diabetic and non-diabetic population, respectively. The risk factor adjusted HRs to predict CVD, except for HDL-C, TG and TG/HDL-C, were significant for all lipid measures in diabetic males and were 1.39, 1.45, 1.36 and 1.16 for TC, LDL-C, non- HDL-C and TC/HDL-C respectively. In diabetic women, using multivariate analysis, only TC/HDL-C had significant risk [adjusted HR1.31(1.10-1.57)].Among non-diabetic men, all lipid measures, except for TG, were independent predictors for CVD however; a 1 SD increase in HDL-C significantly decreased the risk of CVD [adjusted HR 0.83(0.70-0.97)].In non-diabetic women, TC, LDL-C, non-HDL-C and TG were independent predictors.

There was no difference in the discriminatory power of different lipid measures to predict incident CVD in the risk factor adjusted models, in either sex of diabetic and non-diabetic population.

**Conclusion:**

Our data according to important test performance characteristics provided evidence based support for WHO recommendation that along with other CVD risk factors serum TC vs. LDL-C, non-HDL-C and TC/HDL-C is a reasonable lipid measure to predict incident CVD among diabetic men. Importantly, HDL-C did not have a protective effect for incident CVD among diabetic population; given that the HDL-C had a protective effect only among non- diabetic men.

## Background

Risk of cardiovascular disease (CVD) is 200% higher in diabetic population than non-diabetic individuals [1] and coronary heart disease (CHD) is the leading cause of death among diabetic patients [2,3]. Furthermore, as acknowledged by Haffner et al, type 2 diabetic patients without prior myocardial infarction (MI) have as high a risk of MI as non-diabetic patients with prior MI [4]. The increased risk of CHD in diabetic patients is partly attributable to higher prevalence of hypertension, obesity, cigarette smoking and dyslipidemia [5]. It is well known that type 2 diabetes is associated with high prevalence of dyslipidemia, including elevated triglycerides (TG) and reduced high-density lipoprotein cholesterol (HDL-C) and increased low-density lipoprotein cholesterol (LDL-C) particle with altered composition [6]. Diabetes is often associated with many other atherogenic lipoprotein abnormalities characterized by elevated very low density lipoprotein (VLDL), intermediate density lipoprotein (IDL) and chylomicron, and non-HDL-C, including all apolipoprotein B containing atherogenic lipoproteins, has been reported to be superior to LDL-C in predicting incident cardiovascular disease (CVD) in diabetic patients [7,8]. However, total cholesterol (TC)/HDL-C was the best predictor of CVD in diabetic males in the Health Professionals' Follow-up Study [8]. Hence, the relative role of various lipid measures in determining CVD risk in diabetic patients is still a subject of debate [9].

The Tehran Lipid and Glucose Study (TLGS) is a population based study conducted on a representative Iranian urban population, residents of the capital city, Tehran [10]. This Iranian population has a high prevalence and incidence of type 2 diabetes, and Iranian men and women with diabetes have more than 1.7 and 2.5 times risk of incident CVD respectively, independent of traditional risk factors [11-13]. Furthermore, to our knowledge, no previous study has directly compared the predictive power of lipid measures in type 2 diabetic patients among a Middle Eastern population, which has the highest rate of type 2 diabetes predicted by 2030 [14]. Hence, considering the ethnical differences in CVD risk factors and cultural/lifestyle practices, the aim of this study is to evaluate the ability of different lipid measures head-to head for predicting CVD risk in Iranian men and women with and without type 2 diabetes using important performance measures of prediction models including model discrimination (evaluated by C index) [15], a statistic has rarely been applied in the similar studies.

## Methods

### Study population

Subjects in this study were drown among participants of the TLGS, a prospective study to determine the risk factors and outcomes for non-communicable disease. TLGS has been performed on a representative sample of 15005 people aged 3 years and over, residents of district-13 of Tehran; subjects were categorized into the cohort and intervention groups, the latter to be educated for implementation of life style modifications [10,16].

From this overall group, 7614 individuals (1358 diabetic and 6256 non-diabetic subjects) aged 30 years old and over were evaluated in a cross-sectional phase of TLGS (February 1999 to August 2001). Subjects with a history of CVD at baseline (n = 518) were excluded, leaving 7096 individuals; of whom 1021 diabetic (men = 413, women = 608) and 5310 non-diabetic subjects (men = 2317, women = 2993) were followed up March 2009 with a median of 8.6 years (response rate 6331/7096 ≈ 89%). The ethical committee of the Research Institute for Endocrine Sciences approved this study and informed written consent was obtained from all subjects.

### Clinical and laboratory measurements

Using a pretested questionnaire, a trained interviewer collected information which included demographic data, past medical history and family history of CVD, consumption of antihypertensive and lipid lowering drugs and smoking behavior. Subjects were also questioned about past history of diabetes mellitus and taking of any anti-diabetic drugs. Weight was measured, while subjects were minimally clothed without shoes, using digital scales (Seca 707, Seca Corp., Hanover, MD; range 0.1-150 kg) and recorded to the nearest 100 g. Height was measured in a standing position without shoes, using tape meter with shoulders in normal alignment. Body mass index (BMI) was calculated as weight (kg) divided by square of height (m^2^). Waist circumference (WC) was measured at umbilical level, using an unstretched tape meter, without any pressure to body surface and hip circumference (HC) at the maximal level over light clothing. Waist to hip ratio (WHR) was calculated as WC (cm) divided by HC (cm). Two measurements of systolic and diastolic blood pressure (SBP and DBP, respectively) were taken using a standardized mercury sphygmomanometer (calibrated by the Iranian Institute of Standards and Industrial Researches) on the right arm, after a 15 minute rest in a sitting position; mean of the two measurements was considered as subject's blood pressure. A blood sample was drawn between 7:00 and 9:00 AM from all study participants after 12-14 h overnight fasting. All the blood analyses were done at the TLGS research laboratory on the day of blood collection. Plasma glucose was measured using an enzymatic colorimetric method with glucose oxidase. The standard oral glucose tolorance test (OGTT) was performed for all participants not on glucose-lowering drugs. TC was assayed using enzymatic colorimetric method with cholesterol esterase and cholesterol oxidase. HDL-C was measured after precipitation of the apolipoprotein B-containing lipoproteins with phosphotungstic acid. TG was assayed using an enzymatic colorimetric method with glycerol phosphate oxidase. These analyses were performed using commercial kits (Pars Azmoon Inc., Tehran, Iran) and a Selectra 2 auto analyzer (Vital Scientific, Spankeren, The Netherlands). In subjects whose TG concentration was below 4.52 mmol/L, LDL-C was calculated from serum TC, TG and HDL-C concentration, using the Friedewald formula [17]. Non-HDL-C was calculated by subtracting HDL-C from TC; TC/HDL-C and TG/HDL-C were calculated by dividing TC and TG to HDL-C respectively. The intra- and inter-assay coefficients of variation (CV) were both 2.2% for glucose. For both total and HDL-Cholestrol, intra- and inter-assay CVs were 0.5 and 2% respectively. Intra- and inter-assay CVs were 0.6 and 1.6% for TG respectively [10,16].

### CVD outcome

Details of the collection of cardiovascular outcome data have been published elsewhere [18]. To summarize, each participant was followed up for any medical event annually by phone calls. They were asked for any medical condition by a trained nurse and then a trained physician collected complementary data regarding that event during a home visit and by acquisition of data from medical files. The collected data were then evaluated by an outcome committee consisting of an internist, an endocrinologist, a cardiologist, an epidemiologist and other experts, when needed, to assign a specific outcome for every event. In this study, our desired events were the first CVD events, including definite MI [with diagnostic electrocardiogram (ECG) and biomarkers], probable MI (positive ECG findings plus cardiac symptoms or signs plus missing biomarkers or positive ECG findings plus equivocal biomarkers [19], unstable angina (new cardiac symptoms or changing symptom patterns and positive ECG findings with normal biomarkers), angiographic proven CHD, stroke (as defined by a new neurological deficit that lasted more than 24 h) and death from CVD.

### Definition of terms

Diabetes mellitus was defined as fasting plasma glucose (FPG) ≥ 7 mmol/L, 2 hours plasma glucose ≥ 11.1 mmol/L, current use of anti-diabetic drugs [20] or a positive answer to the question "have you ever been told by a doctor that you have diabetes (high blood sugar disease)?" A positive family history of premature CVD reflected any prior diagnosis of CVD by a physician in any female first-degree relative under 65 years old, and any male first-degree relative aged 55 years. Smoking status included current regular or occasional use of cigarettes. According to the World Health Organization (WHO) definition, menopause was defined as absence of spontaneous menstural bleeding for more than 12 months, for which no other pathologic or physiologic cause could be determined [21].

### Statistics

Mean (standard deviation: SD) values for continous and frequencies (%) for categorical variables of the baseline characteristics including lipid measures are expressed for diabetic and non-diabetic participants, with and without CVD. Since FPG, TG, and TG/HDL-C had skewed distribution they are shown as median (interquartile range). Comparison of baseline characteristics between subjects with and without CVD was done by student's t-test for continuous variables, chi-square test for categorical variables and Mann-Whitney test for skewed variables.

Cox proportional hazard model was used to study the association of lipid measures with CVD outcome. Follow up duration was defined as the period between entrance to study and the end points; end point was considered as the first CVD event and censoring which was defined as leaving the residence area, loss to follow up or non-CVD death or March 2009. To select covariates to be included in the multivariate Cox models, univariate analysis was used for each candidate covariate (age, family history of premature CVD, intervention group, WHR, SBP, DBP, FPG, smoking, aspirin use, antihypertensive and lipid lowering drugs in both genders and menopause status in women); following this, each covariate that had a P value less than 0.2 in the univariate analysis, was selected to be included in a stepwise backward (P remove ≤ 0.1) multivariate Cox regression analysis. In diabetic men, final covariates were age, FPG, SBP and lipid lowering drugs; in diabetic women these were WHR, FPG, SBP, lipid lowering drugs use, family history of premature CVD and menopause status. Among non-diabetic men, final covariates were age, family history of premature CVD, WHR, SBP and aspirin; in non-diabetic women those were age, WHR, DBP, smoking status, antihypertensive drug use and positive family history of premature CVD. Each final model included these covariates plus one of the lipid measures. Adjusted hazard ratios (HRs), with 95% confidence intervals (CI), were calculated for every 1 SD increase in the value of each lipid measures.TG and TG/HDL were log-transformed and their HRs were calculated for 1 DS increase in log-transformed TG and TG/HDL. The proportional hazards assumption in the Cox model was assessed with the Schoenfild residual test and all proportionality assumptions were appropriate.

To determine the goodness of fit of the predictive models, the Akaike Information Criterion (AIC), as a statistical estimate of the trade-off between the likelihood of a model against its complexity was used. A lower value of AIC indicates a better model fit [22]. The discrimination ability of the models was calculated using the C index [15]. A value of 1 denotes perfect discrimination and a value of 0.5 is no better than chance. The C indices of multivariate Cox models were compared using the "somersd" STATA command which calculated confidence interval for the Harrell's C index using jackknife variance estimation [23]. The STATA software (version 10) was used for data analysis and P-values ≤ 0.05 were considered statistically significant.

## Results

The study sample consisted of 1021 diabetic individuals (men = 413, women = 608) and 5310 non-diabetic subjects (men = 2317, women = 2993) with mean age of 54.8 and 45.8 year, respectively. Among the diabetic population, of 1021 diabetic subjects 161 cases (15.7%) were identified only by the positive response to the relevant question at baseline. There was no significant difference between subjects followed and those non-followed, in age, CVD risk factors and lipid markers or indices. During a median follow up of 8.6 years, 189 (men = 91, women = 98) and 263(men = 169, women = 94) first CVD events occurred, in diabetic and non-diabetic population, respectively.

Baseline characteristics in diabetic and non-diabetic, with and without CVD events, are summarized in table 1. In diabetic individuals, in comparison with subjects without CVD, those with CVD were older and had significantly higher WHR, SBP, DBP, FPG, TC, LDL-C, TG, non-HDL-C, TC/HDL-C and TG/HDL and higher rate of aspirin, lipid-lowering and antihypertensive drugs consumption; similarly, non-diabetic subjects with CVD were older and had higher WHR, SBP, DBP, FPG and all lipid parameters, but for HDL-C which had lower values in those with CVD. Using aspirin, antihypertensive and lipid lowering drugs were higher in non-diabetic subjects with CVD. Furthermore, the prevalence of smoking and family history of premature CVD was significantly higher in non-diabetic individuals with CVD.

**Table 1:** General Characteristics of diabetic and non-diabetic subjects with and without cardiovascular disease

	**With diabetes**			**Without diabetes**		
		
	**without CVD (n = 832)**	**with CVD (n = 189)**	**P Value**	**without CVD (n = 5047)**	**with CVD (n = 263)**	**P Value**
	
Age	53.9 ± 11.2	58.5 ± 9.6	<0.001	45.2 ± 11.7	56.9 ± 10.7	<0.001
FH premature CVD (%)	18.6	23.8	0.10	15.6	21.4	0.01
Cigarette smoking (%)	10.2	12.7	0.32	14.7	23.7	<0.001
Aspirin use (%)	16.7	24.7	0.01	10.6	20.2	<0.001
Lipid-lowering drug use (%)	8.4	17.0	<0.001	2.5	4.9	0.01
Antihypertensive drug use(%)	16.4	29.1	<0.001	5.5	20.2	<0.001
BMI (kg/m2)	28.8 ± 4.9	28.9 ± 4.1	0.70	27.1 ± 4.5	27.7 ± 4.6	0.03
WHR	0.93 ± 0.08	0.96 ± 0.07	<0.001	0.87 ± 0.08	0.93 ± 0.07	<0.001
SBP (mmHg)	131.4 ± 21	143.4 ± 25	<0.001	118.9 ± 17.8	133.9 ± 22.8	<0.001
DBP (mmHg)	82.0 ± 10	85.6 ± 14	0.001	78.2 ± 10.4	83.3 ± 13.3	<0.001
FPG (mmol/L)	7.2(5.8-9.6)	8.5(6.9-11.8)	<0.001	4.9(4.6-5.3)	5.1(4.7-5.4)	0.002
TC (mmol/L)	5.9 ± 1.2	6.4 ± 1.3	<0.001	5.5 ± 1.1	5.9 ± 1.2	<0.001
LDL-C (mmol/L)	3.7 ± 0.99	4.1 ± 1.11	<0.001	3.5 ± 0.95	3.9 ± 1.05	<0.001
HDL-C (mmol/L)	1.07 ± 0.28	1.03 ± 0.27	0.07	1.09 ± 0.28	1.05 ± 0.27	0.03
TG (mmol/L)	2.2(1.6-3.2)	2.5(1.8-3.4)	0.005	1.7(1.1-2.4)	1.8(1.4-2.7)	<0.001
Non-HDL-C (mmol/L)	5.0 ± 1.2	5.3 ± 1.3	<0.001	4.4 ± 1.1	5.0 ± 1.1	<0.001
TG/HDL-C	2.1(1.4-3.3)	2.3(1.7-3.8)	0.006	1.5(0.9-2.4)	1.8(1.2-2.9)	<0.001
TC/HDL-C	5.8 ± 1.9	6.6 ± 2.2	<0.001	5.3 ± 1.6	5.9 ± 1.7	<0.001

Mean ± SD are shown for continuous variables and P value is calculated with t-test; % is shown for categorical variables with P value according to chi-square; TG, TG/HDL and FPG are shown as median (interquartile range) and P value according to Mann-whitney test. CVD: Cardiovascular disease, FH: family history, BMI: body mass index, WHR: waist to hip ratio, SBP: systolic blood pressure, DBP: diastolic blood pressure, FPG: fasting plasma glucose, TC: total cholesterol, LDL-C: Low-density Lipoprotein, HDL-C: High density lipoprotein, TG: triglyceride.

Table 2 and table 3 present HRs of a 1 SD increase in each lipid marker or index for first CVD event, in diabetic and non-diabetic men and women, respectively. In diabetic men, in age adjusted Cox proportional hazard models, there was positive association between TC, LDL-C, non-HDL-C, TG, TC/HDL-C, and TG/HDL-C with CVD events. After further adjustment for SBP, FPG and lipid-lowering drugs, the associations of these lipids with CVD attenuated, but were still significant for TC, LDL-C, non-HDL-C, TC/HDL-C. Furthermore, a 1 SD increase in log-transformed TG increased marginally the risk of CVD (≈ 23%, p = 0.06). In men, a 1 SD increase of lipid measures increased risk of CVD events from 16% to 45% in the full adjusted models, depending on the lipid measures. In diabetic women, a 1 SD increase of TC, LDL-C, non-HDL-C, TG, TC/HDL-C and TG/HDL-C resulted in a significant risk of incident CVD in age adjusted model, however; a 1 SD increase in HDL-C decreased the risk of CVD ≈ 19% [0.81(0.65-1.01), P = 0.06]. Considering multivariate analysis, only TC/HDL-C was significantly associated with CVD event [1.31(1.10-1.57), P = 0.002], albeit a 1 SD increase in LDL-C and non-HDL-C resulted in an 18% increased risk of CVD. The non significant association of LDL-C and non-HDL-C in diabetic women with incident CVD might be partly attributed to the limited statistical power. With our sample size and α of 0.05, we had the statistical power to detect an HR of 1.20 and 1.30 per 1 SD increment of 30% and 74% respectively, in diabetic women with incident CVD.

**Table 2:** Hazard ratios and discrimination of lipid measures for predicting cardiovascular events in diabetic men and women

		**Age adjusted model**		**Multivariate adjusted model**			
			
	**SD (mmol/L)**	**Hazard ratio (95%CI)**	**P value**	**Hazard ratio (95%CI)**	**P value**	**C index (95% CI)**	**AIC**
	
**men**							
TC	1.18	1.46(1.21-1.75)	<.001	1.39(1.15-1.70)	0.001	0.70(0.65-0.75)	994
LDL-C	0.96	1.47(1.18-1.83)	<.001	1.45(1.16-1.83)	0.001	0.70(0.65-0.76)	804
HDL-C	0.26	0.90(0.71-1.12)	0.36	0.91(0.72-1.16)	0.47	0.68(0.62-0.73)	989
Non-HDL-C	1.16	1.45(1.20-1.74)	<.001	1.36(1.14-1.63)	0.001	0.70(0.65-0.75)	980
TG	0.58*	1.27(1.03-1.57)	0.02	1.23(0.99-1.53)	0.06	0.68(0.63-0.74)	1001
TC/HDL-C	2.20	1.18(1.04-1.34)	0.008	1.16(1.01-1.33)	0.02	0.69(0.64-0.74)	986
TG/HDL-C	0.71*	1.22(0.99-1.49)	0.05	1.18(0.96-1.47)	0.11	0.68(0.62-0.73)	987
	
**women**							
TC	1.29	1.32(1.11-1.57)	0.001	1.13(0.93-1.37)	0.19	0.73(0.68-0.77)	1137
LDL-C	1.04	1.33(1.11-1.59)	0.002	1.18(0.97-1.44)	0.09	0.73 (0.68-0.78)	980
HDL-C	0.28	0.81(0.65-1.01)	0.06	0.82(0.66-1.02)	0.08	0.73(0.68-0.77)	1126
Non-HDL-C	1.28	1.37(1.16-1.62)	<.001	1.18(0.98-1.42)	0.07	0.73(0.69-0.78)	1126
TG	0.51*	1.22(1.01-1.48)	0.03	1.08(0.89-1.33)	0.4	0.72(0.67-0.77)	1138
TC/HDL-C	1.80	1.44(1.24-1.68)	<.001	1.31(1.10-1.57)	0.002	0.74(0.70-0.79)	1121
TG/HDL-C	0.62*	1.30(1.07-1.59)	0.007	1.16(0.95-1.42)	0.12	0.72(0.69-0.78)	1127

SD: standard deviation.TC: total cholesterol, LDL-C: low density lipoprotein cholesterol, HDL-C: high density lipoprotein cholesterol, TG: triglyceride, CI: confidence interval. Hazard ratios indicate the increase risk for a 1 SD increase of each lipid parameter. Multivariate models were adjusted for age, systolic blood pressure (SBP), Fasting plasma glucose (FPG) and lipid lowering drug use in men and were adjusted for SBP, FPG, waist to hip ratio, lipid lowering drug use, menopause status and family history of premature cardiovascular disease in women. AIC (Akaike Information Criterion) is a statistical estimate of the trade-off between the likelihood of a model against its complexity; a lower value of AIC indicates a better model fit. The discrimination ability of the models was calculated using the C index. A value of 1 denotes perfect discrimination and a value of 0.5 is no better than chance. *SD of log-transformed.

**Table 3:** Hazard ratios and discrimination of lipid measures for predicting cardiovascular events in non-diabetic men and women

		**Age adjusted model**		**Multivariate adjusted model**			
			
	**SD (mmol/L)**	**Hazard ratio (95%CI)**	**P value**	**Hazard ratio (95%CI)**	**P value**	**C index (95% CI)**	**AIC**
	
**men**							
TC	1.07	1.25(1.08-1.44)	0.002	1.21(1.04-1.41)	0.009	0.76(0.73-0.80)	2389
LDL-C	0.91	1.24(1.07-1.44)	0.004	1.23(1.06-1.44)	0.006	0.77(0.74-0.80)	2236
HDL-C	0.24	0.83(0.71-0.97)	0.026	0.83(0.70-0.97)	0.02	0.77(0.73-0.80)	2374
Non-HDL-C	1.07	1.30(1.13-1.50)	<0.001	1.27(1.10-1.48)	0.001	0.77(0.74-0.80)	2369
TG	0.54*	1.19(1.03-1.37)	0.013	1.13(0.96-1.32)	0.12	0.76(0.73-0.79)	2393
TC/HDL-C	1.71	1.28(1.12-1.46)	<0.001	1.29(1.12-1.48)	<0.001	0.77(0.74-0.80)	2368
TG/HDL-C	0.68*	1.25(1.07-1.47)	0.005	1.20(1.01-1.43)	0.03	0.76(0.73-0.80)	2375
	
**women**							
TC	1.17	1.29(1.08-1.55)	0.005	1.22(1.01-1.48)	0.03	0.84(0.81-0.87)	1313
LDL-C	0.98	1.30(1.06-1.59)	0.01	1.22(0.99-1.49)	0.05	0.84(0.80-0.87)	1206
HDL-C	0.28	0.95(0.78-1.17)	0.68	1.00(0.81-1.24)	0.94	0.84(0.80-0.87)	1317
Non-HDL-C	1.18	1.30(1.08-1.56)	0.004	1.22(1.01-1.48)	0.03	0.84(0.81-0.87)	1313
TG	0.52*	1.36(1.10-1.68)	0.004	1.27(1.01-1.59)	0.03	0.84(0.81-0.87)	1313
TC/HDL-C	1.63	1.24(1.04-1.47)	0.01	1.17(0.97-1.41)	0.09	0.84(0.81-0.87)	1314
TG/HDL-C	0.66*	1.30(1.05-1.62)	0.01	1.21(0.96-1.52)	0.10	0.84(0.80-0.87)	1314

SD: standard deviation, TC: total cholesterol, LDL-C: low density lipoprotein cholesterol, HDL-C: high density lipoprotein cholesterol, TG: triglyceride, CI: confidence interval. Hazard ratios indicate the increase risk for a 1 SD increase of each lipid parameter. Multivariate models were adjusted for age, family history of premature CVD, WHR, SBP and aspirin in men and were adjusted for age, WHR, DBP, smoking status, antihypertensive drug use and family history of premature CVD age, WHR, DBP, smoking status, antihypertensive drug use and positive family history of premature CVD in women. AIC (Akaike Information Criterion) is a statistical estimate of the trade-off between the likelihood of a model against its complexity; a lower value of AIC indicates a better model fit. The discrimination ability of the models was calculated using the C index. A value of 1 denotes perfect discrimination and a value of 0.5 is no better than chance. *SD of log-transformed.

Among non-diabetic men, in age adjusted models, all lipid measures were significantly associated with risk of CVD. After further adjustment for risk factors, all of the lipid measures remained significant predictors of CVD except for TG. The multivariate HRs of a 1 SD increase in TC, LDL-C, non-HDL-C, TC/HDL-C and TG/HDL-C for predicting CVD were 1.21, 1.23, 1.27, 1.29 and 1.20 respectively. A 1 SD increase of HDL-C decreased risk of CVD by 17% in both models in non-diabetic men. In women without diabetes, a 1 SD increase of TC, LDL-C, non-HDL-C, TG, TC/HDL-C and TG/HDL-C were associated with 24% to 36% increased risk of CVD (all P value < 0.05) depending on lipid markers, in age adjusted models. However, after further adjustment for risk factors, TC, LDL-C, non-HDL-C and TG were significantly associated with incident CVD. In non-diabetic women, HDL-C did not show any association with CVD risk in both models.

Regarding the discriminatory power of the models, we found that multivariate models with different lipid measures had relatively the same C index in each of the genders in both diabetic and non-diabetic (P > 0.05 for differences in C indices).

According to AIC, LDL-C had the best fitness in multivariate models in diabetic men, followed by non-HDL-C, TC/HDL-C and TC. In women with diabetes, the LDL-C highlighted the best fitted multivariate model, followed by TC/HDL-C. Similarly in non-diabetic men, the smallest AIC in the multivariate models belonged to the LDL-C model followed by TC/HDL-C. In non-diabetic women, according to AIC, the LDL-C model was the fittest model, however, the TC, non-HDL-C, TG models showed similar AICs, which were more than LDL-C.

Finally, we also evaluated the predictive value of TC, LDL-C, non-HDL-C and TC/HDL-C for incident CVD in diabetic participants with TG levels ≥ 2.26 and <2.26 mmol/L in sex pooled analysis. The associations of the lipid measures with CVD risk were stronger among diabetic participants with lower TG levels than those with higher TG levels (Table 4). However, tests for interaction of TG with mentioned lipids were all non significant (all P value > 0.05).

**Table 4:** Adjusted Hazard ratios for cardiovascular disease in diabetic subjects with Triglyceride level less and more than 2.26 mmol/L

	Triglyceride ≥ 2.26 mmol/L		Triglyceride < 2.26 mmol/L	
		
	Hazard ratio (95% CI)	P value	Hazard ratio (95% CI)	P value
TC	1.23(1.03-1.45)	0.01	1.40(1.04-1.89)	0.02
LDL-C	1.30(1.07-1.57)	0.007	1.39(1.07-1.81)	0.01
Non-HDL-C	1.24(1.04-1.47)	0.01	1.46(1.09-1.97)	0.01
TC/HDL-C	1.18(1.05-1.33)	0.004	1.74(1.19-2.55)	0.004

TC: total cholesterol, LDL: low density lipoprotein cholesterol, HDL-C: high density lipoprotein cholesterol. Hazard ratios indicate the increase risk for a 1 standard deviation increase of each lipid measures; adjusted for age, sex, systolic blood pressure, fasting plasma glucose, lipid lowering drug use, waist to hip ratio and positive family history of premature cardiovascular disease. The method of selection of the covariates is mentioned in statistics.

## Discussion

In this population based prospective cohort of diabetic and non-diabetic Iranian men and women, we examined different lipid measures as predictors of CVD events using multivariable Cox regression analysis. In diabetic men, TC, LDL-C, non-HDL-C, and TC/HDL-C remained as independent predictors of incident CVD. Although the differences were small in discriminatory terms (as assessed by C index), the LDL-C model had the better fitted model (as assessed by AIC) followed by the non-HDL-C, TC/HDL-C, TC and TG models. In diabetic women, after adjustment for CVD risk factors, only TC/HDL-C resulted in a significant risk for CVD. Similarly in diabetic women, although the differences were small in discriminatory terms, LDL-C had the better fitted model followed by TC/HDL-C model. Among non-diabetic men, all lipid measures, except for TG, were independent predictors for CVD; a 1 SD increase in HDL-C however, significantly decreased the risk of CVD, with almost same discriminatory power. In female non-diabetic population, TC, LDL-C, non-HDL-C and TG showed significant association with CVD event with the same discrimination in full adjusted analysis.

A large body of evidence has shown that high serum TC and LDL-C are risk factors for CVD in the diabetic and non-diabetic population [24,25]. The current study yielded the same discriminatory power for TC and LDL-C in both genders of diabetic and non-diabetic population, although based on the AIC; the LDL-C model had better risk prediction. In the Asia Pacific Cohort Studies Collaboration for each increase of a 1 SD in TC, participants with diabetes had a 41% (95%CI: 23-63%) greater risk of CHD in age and sex adjusted analysis [26]; the corresponding estimate for male participants in our study was 39% (15%-70%). Indeed, the predictive power of the Framingham coronary risk score does not change when LDL-C is used in place of TC [27]. When plasma TG levels are not so high, the plasma TC is generally correspondent with LDL-C level; therefore, in a diabetic population with a high prevalence of hypertriglyceridemia (in our study 10.7% of the whole diabetic population have TG ≥ 4.52 mmol/L) the use of LDL-C might be better than that of TC [28].

Previous studies have reported the role of non-HDL-C in prediction of CVD risk among diabetic and non-diabetic population, as a simple and reproducible lipid measure which does not need fasting state for its measurement [7,8,29,30]. Our finding demonstrated that among the non-diabetic population, non-HDL-C was a significant predictor of incident CVD in both genders; similar to the association shown among non-diabetic Arab community [30]. In diabetic population strong evidence for its superiority over TC, LDL-C (as the main lipid target) and TC/HDL-C is not yet available [31]. In diabetic men of the Health Professionals' Follow-up Study, comparisons of nested models highlighted that non-HDL-C, but not ApoB, add significantly to the prediction of CVD risk beyond LDL-C; nevertheless, in this study TC/HDL-C was the best predictor of CVD [8]. Furthermore in the Strong Heart Study, the HRs for the highest tertile of non-HDL-C in men and women with diabetes were higher than those for either LDL-C or TG per se in both genders and were higher than the TC/HDL-C in women, although the CIs overlapped [7]. In our data analysis, TC/HDL-C and non-HDL-C had same discriminatory power and relatively the same model fitness in both genders of diabetic population; although non-HDL-C in men and TC/HDL-C in women had the least AIC (except LDL-C). Hence, the similarity of the C index between TC/HDL-C and non-HDL-C to predict incident CVD in our study indicates that, at least in type 2 diabetic patients the difference is not clinically important as reported by Holman et al in the U.K. Prospective Diabetes Study (UKPDS) [32]. In agreement with the findings of our study, non-HDL-C was associated with CVD risk among diabetic subjects independent of their TG levels as shown in other diabetic cohorts [7,8]. However, in contrast to the Strong Heart Study, the significant risk of TC/HDL-C in prediction of incident CVD was not affected by TG levels [7].

In this study, among the diabetic population, HDL-C in both genders was not associated with incident CVD, although there was significant protective effect of HDL-C in non-diabetic men. Oant et al. showed that in the general Turkish adult population, a 12 mg/dl increase of HDL-C in men, decreased the risk of CHD by 20%, whereas HDL-C did not protect the development of CHD in women [33]; similarly ≈ 9 mg/dl (0.24 mmol/L) increase in HDL-C decreased by the 17% risk of CVD in our non-diabetic men, but not in non-diabetic women. Our data analyses support the issue that the atheroprotective effect of HDL-C may be deficient in subjects with type 2 diabetes mellitus [33]. As acknowledged by Kuntush et al, the compositional modification of the HDL-C lipid core and conformational change of ApoA-1 might lead to functional alteration of the HDL-C particle in type 2 diabetes[34]. In our results, TG was not associated with CVD in diabetic females and has a marginal association in diabetic males. In line with current results, HDL-C and TG were not associated with incident CHD in diabetic women of Nurses' Health Study during 10 years of follow-up [29]. Schulze et al suggested that the risk of TG and protective effect of HDL-C were present only among women with low HbA1C and this association was not present among those with poorly controlled diabetes [29]. Furthermore, in diabetic adult men of the Health Professionals' Follow-up Study and the Strong Heart Study the serum TG was an independent predictor of CVD risk [7,8].

In the current study, TG/HDL-C did not show any association with CVD in both genders of diabetic population and also in non-diabetic women. However, as recently showed in general men population, TG/HDL-C remained as an independent predictor of incident CVD among non-diabetic men population [35].

There are several points that should be considered when considering the results of this study. First, we measured TC, HDL-C and TG only once at the baseline per subject, thus the potential bias resulting from regression dilution of TG and HDL-C could not be ignored [36]; additionally, given the significant intra-individual variation in fasting TG level, it might be possible that a proportion of participants who had a high concentration of TG are falsely classified as low, and vice versa; hence, attenuating the true relative risk of the lipoprotein. Second, ApoA-1 and ApoB were not measured at the baseline; however, Ingelsson et al in a large population based study highlighted that the overall performance of ApoB/ApoA-1 for prediction of CHD was comparable with that of traditional lipid ratios, and did not result in incremental utility over TC/HDL-C [37]. Third, we did not consider the inflammatory parameters including CRP, fibrinogen and Apo A-1. Onat et al recently showed that in a Turkish population with high prevalence of metabolic syndrome, obesity and hypertriglyceridemia and low HDL-C (a background similar to residents of Tehran [38]) the positive correlation observed between HDL-C and fibrinogen and Apo A-1 might be cited as biological evidence of functional impairment of HDL-C [33]. Fourth, we did not have data regarding the level of glycemic control (i.e. HbA1C) and duration of diabetes; the potent predictors of CVD risk in diabetic patients [39], and hence, the mentioned factors were not controlled in data analysis. Additionally, we had no information regarding type of diabetes, although by including persons aged ≥ 30 years, we distinguished subjects with higher probability of type 2 than type 1 diabetes. Finally, some misclassification of diabetes status may have occurred due to defining new diagnosed diabetes with a single OGTT at baseline and a partial reliance on self-reported history of diabetes. However, the extent of misclassification would have to be extensive to change the results of this study [26].

The strengths of our study include, the population based sample consisting of diabetic men and women, the continuous monitoring for CVD events and comprehensive assessment of several lipid measures using model discrimination and fitness. As pointed by Ingelsson et al, in studies focusing on prediction of incident CVD, an analysis of the comparative performances in terms of discrimination (C index) may be more important than a comparison of relative risk estimates for lipid measures [37]. Furthermore, given the relatively equal performances of different lipid measures, other factors including the costs, availability of assays and educational needs for health care professional, the possibility of obtaining accurate lipid measure in nonfasting state (i.e. TC, TC/HDL-C and non-HDL-C) and the availability of appropriate therapeutic cutoff points should be borne in mind, when considering guiding the choice of lipid measure that should be used in CVD risk prediction [37].WHO recommended TC as the only lipid parameter for CVD risk assessment of individuals detected to have hypertension, diabetes or smoking behavior in low and medium resource settings where it is both time-consuming and costly to measure TC, TG, LDL-C and HDL-C altogether [40].

## Conclusions

Our data according to important test performance characteristic provided evidence based support for WHO recommendation that along with other CVD risk factors serum TC vs. LDL-C, non-HDL-C and TC/HDL-C is a reasonable lipid measure to predict incident CVD among diabetic men of a Middle Eastern cohort. Importantly, HDL-C did not have a protective effect for incident CVD among diabetic population; given that the HDL-C had a protective effect only among non-diabetic men.

## Competing interests

The authors declare that they have no competing interests.

## Authors' contributions

MT participated in the conception and design of the study; interpretation of data and drafting the manuscript. MH participated in statistical analysis and interpretation of data and drafting the manuscript. FH participated in the conception and design of the study and revised the manuscript for important intellectual content and final approval. MS participated in the conception and design and statistical analyses. HH revised the manuscript for important intellectual content. FA participated in its design and coordination. All authors read and approved the final manuscript.
